# Grunting's competitive advantage: Considerations of force and distraction

**DOI:** 10.1371/journal.pone.0192939

**Published:** 2018-02-22

**Authors:** Scott Sinnett, Cj Maglinti, Alan Kingstone

**Affiliations:** 1 Department of Psychology, University of Hawaii at Manoa, Honolulu, Hawaii, United States of America; 2 Department of Psychology, University of British Columbia, Vancouver, British Columbia, Canada; Auburn University, UNITED STATES

## Abstract

**Background:**

Grunting is pervasive in many athletic contests, and empirical evidence suggests that it may result in one exerting more physical force. It may also distract one's opponent. That grunts can distract was supported by a study showing that it led to an opponent being slower and more error prone when viewing tennis shots. An alternative explanation was that grunting masks the sound of a ball being hit. The present study provides evidence against this alternative explanation by testing the effect of grunting in a sport—mixed martial arts—where distraction, rather than masking, is the most likely mechanism.

**Methodology/Principal findings:**

We first confirmed that kicking force is increased when a grunt is performed (Experiment 1), and then adapted methodology used in the tennis study to mixed martial arts (Experiment 2). Lifting the foot to kick is a silent act, and therefore there is nothing for a grunt to mask, i.e., its effect on an opponent’s response time and/or accuracy can likely be attributed to attentional distraction. Participants viewed videos of a trained mixed martial artist kicking that included, or did not include, a simulated grunt. The task was to determine as quickly as possible whether the kick was traveling upward or downward. Overall, and replicating the tennis finding, the present results indicate that a participant's response to a kick was delayed and more error prone when a simulated grunt was present.

**Conclusions/Significance:**

The present findings indicate that simulated grunting may distract an opponent, leading to slower and more error prone responses. The implications for martial arts in particular, and the broader question of whether grunting should be perceived as 'cheating' in sports, are examined.

## Introduction

The accurate perception of an opponent's action is critical for success in nearly all athletic domains. Distracting an opponent can therefore lead to an advantage. Not surprisingly, this knowledge is commonly used by fans to help create a ‘home-team’ edge. For instance, some spectators at professional baseball or basketball games intentionally attempt to distract the rival team from performing their best (e.g., when shooting free throws in basketball home team fans often scream pejorative remarks or wave their arms in an obvious attempt to distract the opposing team’s player). While this type of behavior by the fans is largely accepted, it can be construed as cheating or at the very least an unfair practice when it is the opposing athlete who attempts to distract an opponent. This point has emerged in professional tennis, with some claiming that distracting sounds, this time made by tennis players in the form of grunting, are used as a means of purposely distracting their opponents. Indeed, even some of tennis’ legends (e.g., Martina Navratilova) have made the claim that grunting is essentially cheating. However, only recently has empirical evidence been offered to suggest that grunting actually confers an advantage to the grunter [1, 2, 3, 4].

It is important, however, to distinguish between two possible advantages that grunting may provide: an increase in force or power for the grunter and/or the distraction of an opponent. With regard to the former, the empirical evidence supports the notion that force can be increased if an action is accompanied by a strong exhalation of breath (e.g., a grunt). For instance, Ikai and Steinhaus investigated whether shouting could increase the force applied to a cable tensiometer and showed that force increased by 12% during an isometric forearm flexor task when shouting [5]. Similar increases in grip strength were observed with karate practitioners when grunting (called a kiai), regardless of expertise level [6]. In contrast, Morales et al. [7] failed to observe any significant effect of grunting on maximal dead lifts. However, the participants in that study only performed three deadlifts with grunts and three without. With such a small number of trials and an insensitive successful lift vs. unsuccessful lift measurement, this null result must be treated with caution. This heed is especially warranted as more recent evidence indicates that grunting increases the power of serves and forehands in tennis [2]. Specifically, when striking a ball grunting improved dynamic stroke velocity, isometric muscle force, and the peak electromyographic (EMG) response. In subsequent research, O’Connell et al. [3] tested the robustness of this result by measuring force when collegiate tennis players struck a tennis ball (forehands), while at the same time measuring different breathing maneuvers (forced exhalation or inhalation, grunting, or trying to exhale though a closed airway (the valsalva maneuver)). The results indicated that grunting, as well as strong exhalation, lead to increased force.

Yet, grunts might also yield an advantage by distracting one's opponent. This was recently tested by Sinnett and Kingstone [4]. In this study participants were required to watch video recordings of a tennis player hitting the ball to the left or right side of the court, with the task being to determine the direction of the shot as quickly and accurately as possible. Half of the video clips were accompanied by a simulated grunt. The findings suggested that participants were both slower and less accurate to respond when a simulated grunt accompanied the shot. While the translation from the laboratory to the real-world can at times be difficult for research in the cognitive sciences (see [8]), these findings could have direct implications for the sport. In fact, when adopting the highly conservative estimate that a professional tennis player hits the ball at 50mph, the delay in response time resulting from the grunt translated into a return shot traveling an extra two feet before the opponent (non-grunter) could respond. Furthermore, based on the average number of shots per point [9] and the average number of points per game [10], this drop in accuracy equates to an opponent being wrong-footed by a grunt once per game.

Sinnett and Kingstone [4] suggested that there were two plausible ways that grunting could negatively impact a tennis opponent. One way was that a grunt could mask the sound that is made when a ball is being struck by the racquet. Given that this sound provides the opponent with important information relative to the force with which a ball is struck as well as its spin, both of which may benefit performance, then masking the sound could lead to a reduction in performance. A recent, and dramatic demonstration of this is provided by Farhead and Punt [1]. Participants judged the speed of serves hit by professional tennis players that were presented on a computer screen. These videos were edited such that they either did or did not include grunts. The results indicated that grunting led to serves being judged as having a higher velocity than they actually had. This finding dovetails with a wealth of previous research indicating that sound can have a dramatic effect on the perception of visual events [11, 12, 13, 14]. Nevertheless, it is also worth noting that these past studies, like the present study, involve situational simplifications that will ultimately demand future investigations that narrow the gap between testing and actual environments and participant expertise. For instance, neither the types of grunting nor the expertise of observer have been systematically manipulated.

An alternative possibility to perceptual masking is that the abrupt sound of a grunt distracts an opponent's attention away from the ball being struck (e.g., [15, 16]). This distraction could operate either automatically, as when attention is captured by a door slam or the sound of a sneeze (see for example [17, 18, 19]), or because attempts to ignore an item results in attention being committed to it, as when an annoying sound, such as a water tap dripping in a sink or someone talking loudly on a phone, becomes the focus of attention (see for example [20, 21]).

The aim of the present investigation is to discriminate between these two possibilities by applying, with a mixed martial arts (MMA) situation, a 'divide and conquer' research approach, whereby one seeks to exclude one of the two prevailing explanations [22]. The kiai is a type of 'grunt' commonly used in martial arts such as karate [23, 24]. Critically, unlike the sound of a tennis ball being struck, the act of kicking in martial arts is silent. Therefore, there is nothing that the grunt can mask during the kick. Accordingly, if grunting results in participants’ judgments being less accurate and/or slower, perceptual masking cannot be the reason, i.e., the alternative distraction account is favoured.

## Experiment 1: Materials and methods

As a first step in this investigation it is important to establish that executing the grunting sound of a kiai actually plays a functional role in the key behaviour—kicking—that will be assessed in Experiment 2. If it does increase force then arguably its use may not be construed as 'cheating'. For example, should grunting allow a kicker to hit harder, then the argument that its use is a form of cheating becomes less tenable, even if it distracts an opponent. We return to this issue in the general discussion. Thus, Experiment 1 measured whether grunting increases the force of a kick. Participants were required to kick a heavy bag equipped with an accelerometer. If grunting does lead to more force, then participants should generate greater kick force when grunting.

### Ethics statement

Informed written consent, abiding to and approved by the University of Hawaii at Manoa’s institutional review board (IRB) was obtained prior to participating in the experiment. This study was approved by the University’s IRB.

### Participants

Twenty participants (n = 20) from a local MMA academy (Honolulu, Hawaii) were recruited for this study (18 males and 2 females). Participants ranged in age (20–35y), height (1.68–1.89 m), and weight (66–93 kg). Each participant had at least one year of MMA/Muy Thai training, with several participants having professional competitive experience.

### Stimuli, apparatus & procedure

Each participant kicked a 100 lb. heavy bag. To measure the amount of g-force generated on the bag by each kick a Herman Digital Trainer accelerometer from Pro Sport Technology Limited was attached to the back of the bag (i.e., the opposite side of the bag that was kicked) at the participant’s hip level. The accelerometer provided immediate results which were recorded by the experimenter.

All participants completed two sessions, which took place one week apart at the MMA academy. Each test session commenced approximately 15 minutes after all participants completed a 60-minute Muay Thai/MMA class, and thus they were sufficiently warmed up before the experiment began. Each Muay Thai/MMA class consisted of an initial 10–15 minute warm-up session involving specific Muay Thai drills, followed by alternating periods of instruction (of a specific technique, e.g., striking, defending, footwork, etc.) and drilling (of the learned technique). After the class and before each test session began, participants prepared for kicking the bag with whatever personal approach they had (e.g., breathing method, stance, etc.). Each test session consisted of a grunting and a no grunting condition with their order counterbalanced across participants and sessions. In each session participants were instructed to use their dominant foot to kick the heavy bag (five mid-level kicks) as hard as they could. Participants were given a 30 second break between kicks to avoid fatigue and reset the position of the heavy bag. While participants were permitted to rest longer if they felt fatigued, none did so.

## Results & discussion

**Fig 1:**
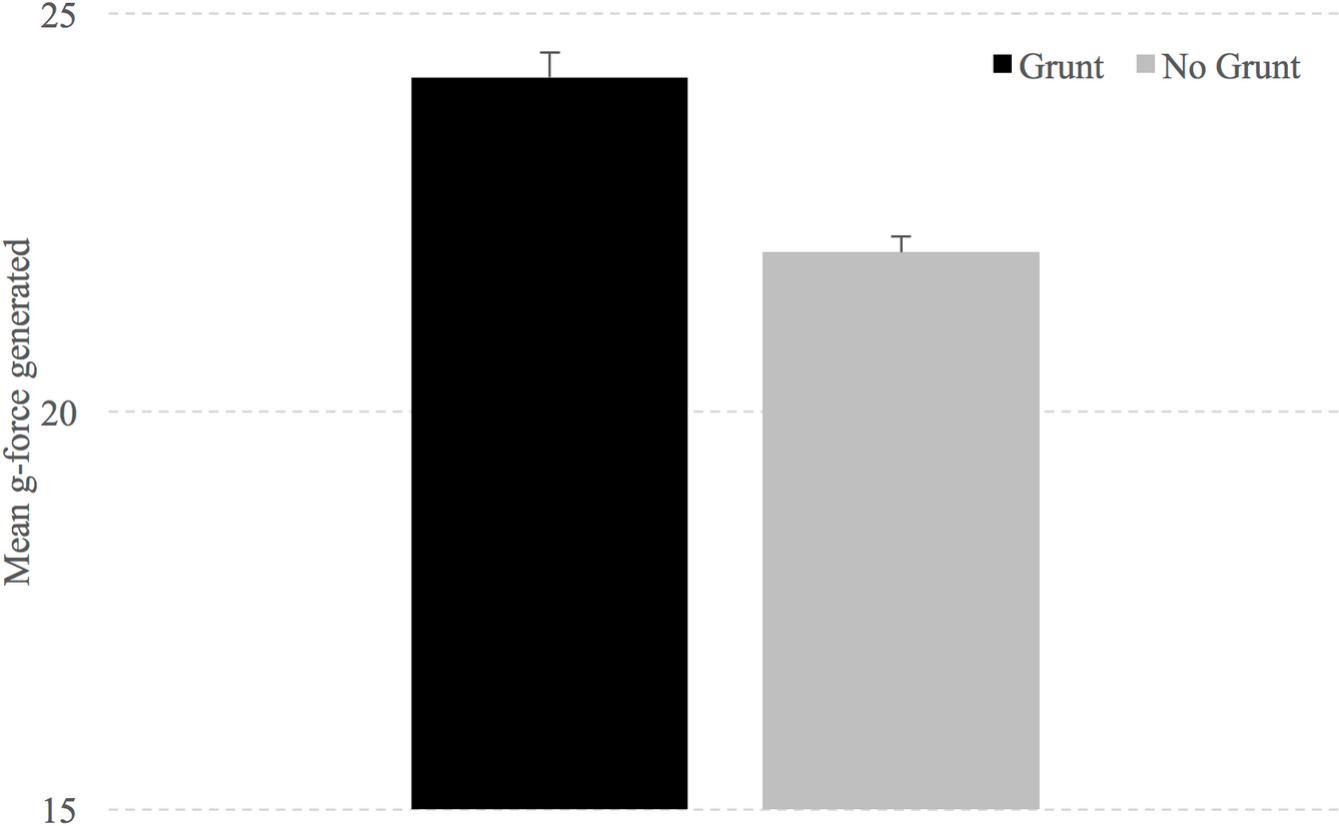
Mean g-force generated while kicking with and without a kiai. Error bars represent standard error of the mean.

Participants were able to generate significantly more g-force while kicking with a grunt than without (see Fig 1). Specifically, participants generated 24.2 g-forces when grunting and 22 g-forces when not grunting (t(19) = 3.371, p = .003), a 9% increase in force.

## Experiment 2: Materials and methods

Given that grunting had a significant effect on kicking performance, Experiment 2 focused on determining whether the effect of simulated grunts observed in Sinnett and Kingstone [4] can be attributed to perceptual masking or attentional distraction. Applying the same methodology to that used in Sinnett and Kingstone, participants here were required to judge as quickly and as accurately as possible the direction of kicks that were either performed with a simulated grunt or not.

### Participants

Twenty-two (n = 22) students (11 male, 11 female) from the University of Hawaii at Manoa were recruited from an undergraduate psychology course (average age 22.6, range 18–47), and received extra credit for their participation. In order to avoid any potential confound regarding past experience, none of the participants had any experience in martial arts or kicking, with the exception of one participant who had taken a kick boxing cardio class (i.e., for exercise purposes). All participants had normal or corrected-to-normal hearing and vision.

### Stimuli, apparatus, and procedure

**Fig 2:**
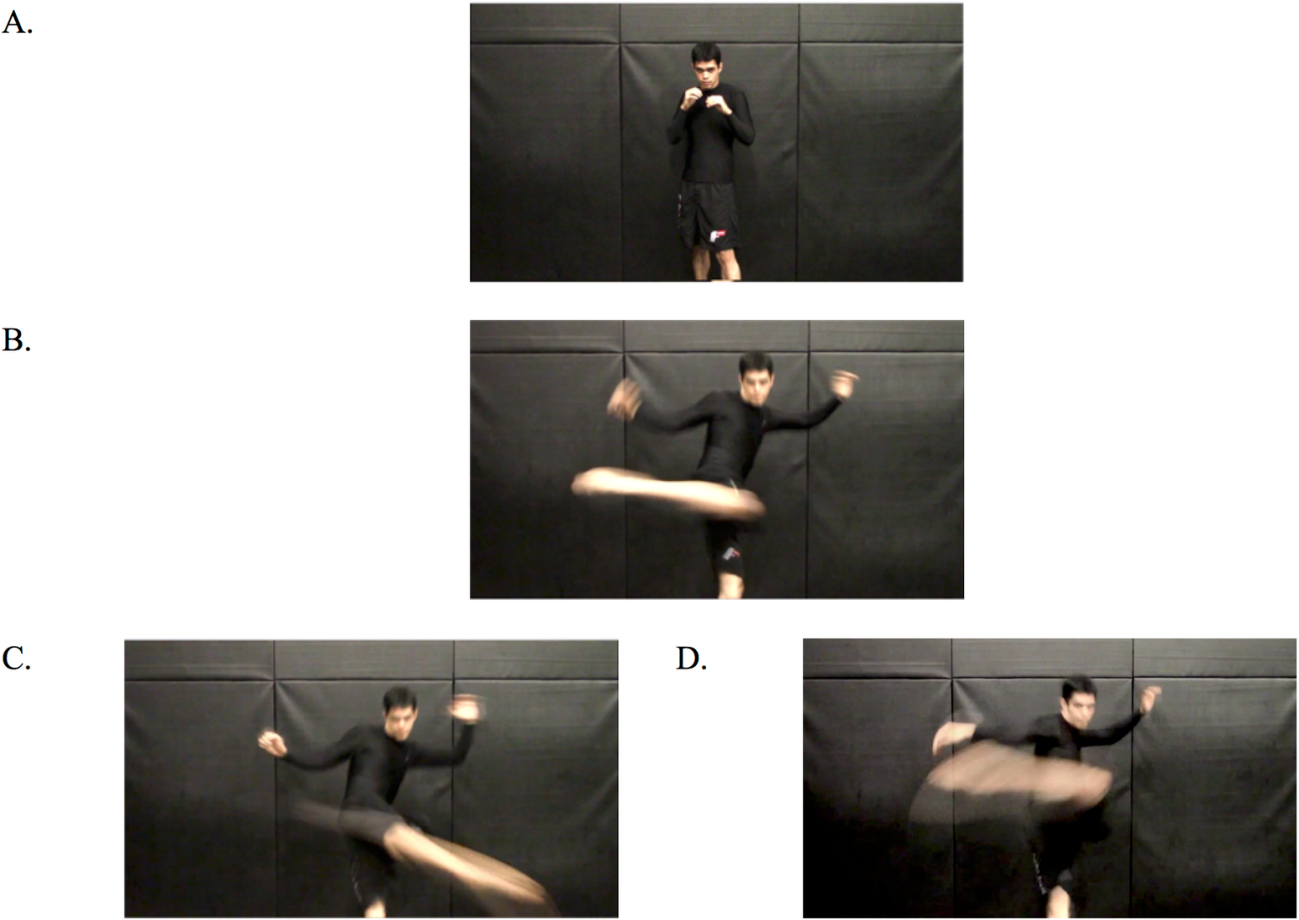
Selected frames depicting the beginning of the video (A), the reorientation point of the kick (B), and examples of low (C) and high (D) kicks. Note, the video ended at the reorientation point (B) in the difficult condition, and 100 ms later in the easy condition (C, D). The individual depicted has given written informed consent (as outlined in the PLOS consent form) to appear in this figure.

The DMDX software (http://www.u.arizona.edu/~jforster/dmdx.htm) was used to present videos and record responses. A video camera was set approximately two meters away and directly in front of a martial artist so that the full body could be recorded in the video clip. The kick height—high or low—was varied. To be included as a stimulus clip, the direction of the kick needed to be concealed until after the reorientation of the kick. The reorientation of the kick is the midway point, after which the kick can either continue upward for a high kick, or reorient and travel downward for a low kick (see Fig 2). The video clips were edited so as to include an equal number of high and low kicks using both the left and right leg equally. There was a total of five video clips for each kick type (total of 20).

Every video was played either with or without a simulated grunt, and ended either at the reorientation of the kick (hard decision) or 100 ms after reorientation of the kick (easy decision, see [4] for the same difficulty manipulation). This resulted in a total of 80 video clips that were presented in a random order (average length of 2043 ms). Computer speakers were used to play the sound at a comfortable volume (approximately 60 dbs). This stimulus configuration ensured that the auditory and visual stimuli appeared to originate from the same central spatial location.

To further match our study with that of Sinnett and Kingstone [4] we used their same standardized auditory stimulus (i.e., an abrupt 500 ms white noise sound burst) to simulate the occurrence of a grunt. While this sound cannot be considered a natural grunt, it does have the benefit of controlling for the many differences related to pitch, length, and amplitude that natural grunts would have. The task required participants to respond as quickly and as accurately as possible indicating the direction of the kick in each video clip. Participants used the M key on a keyboard with their right hand to indicate if they thought the kick was going up, and the Z key on a keyboard with their left hand if they thought the kick was going down (counterbalanced). Each trial began with a fixation cross (2000 ms), followed by the video. Each trial was separated by 2000 ms, with the entire experiment session taking 10–15 minutes. The experiment was conducted in a sound attenuated testing room.

## Results

Analyses of variance with the within-subjects factors of Simulated Grunt (Present vs. Absent) and Difficulty (Hard vs. Easy; i.e., clips ending at the reorientation point vs. clips ending after this point, respectively) were performed on the mean RT and error data.

**Fig 3:**
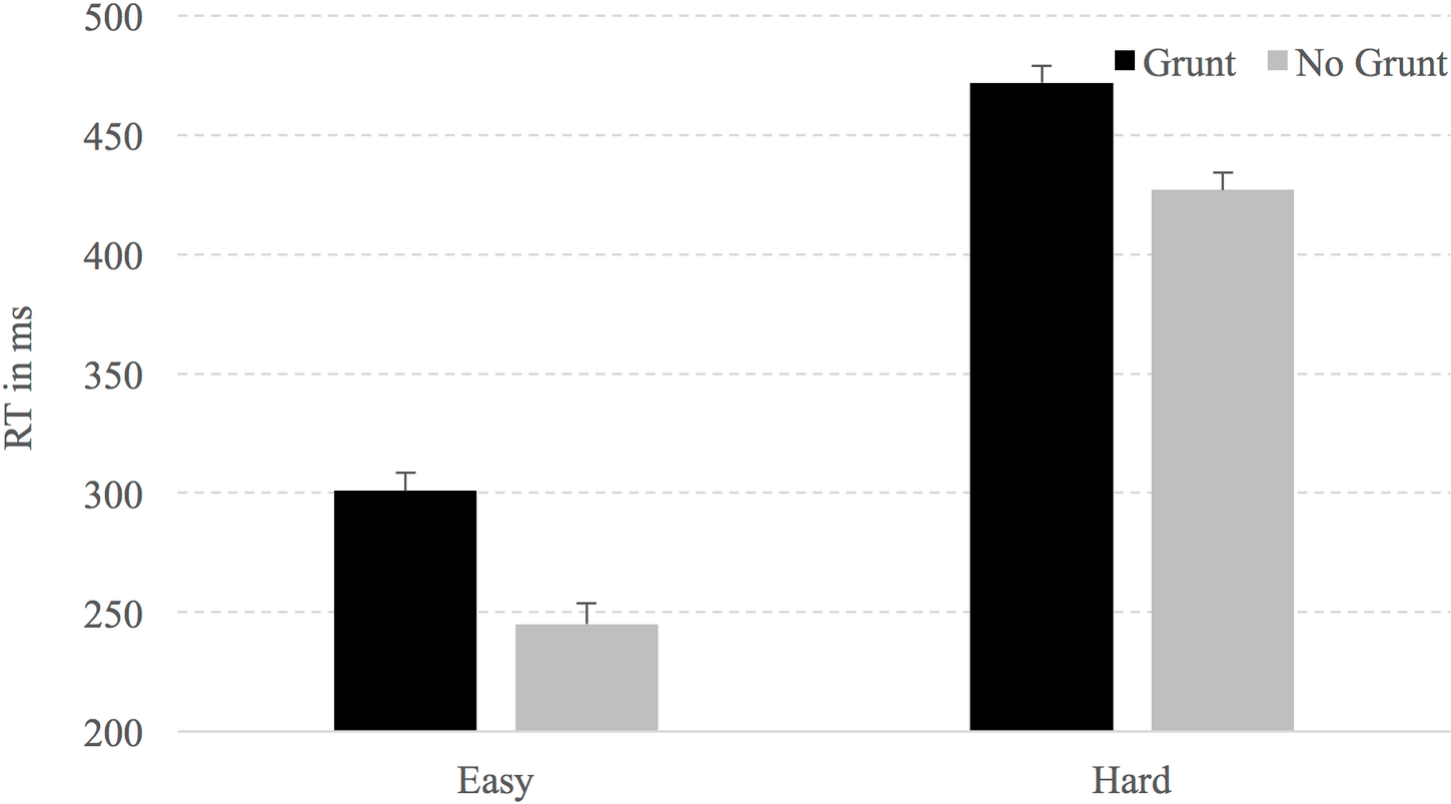
Response latencies for easy and hard conditions. Error bars represent standard error of the mean.

When analyzing response latency a main effect of Difficulty, F(1,21) = 570.4, p < .001 was observed, with RTs to hard clips being much slower than RTs to easy clips (449 ms vs. 273 ms respectively). Similarly, clips with the simulated grunt were responded to more slowly than clips without (387 ms vs. 336 ms, respectively), F(1,21) = 53.3, p < .001. 16.0, p < .001. An interaction was not observed, F(1,21) = .489, p = .49. Planned comparisons (see Sinnett & Kingstone, [4]) revealed that when the simulated grunt was present and the video stopped at the moment of reorientation of the kick (hard decision), the participants were 45 ms slower to respond to the direction of the kick (472 ms, SE = 7.5, versus 427 ms, SE = 7.2; t(21) = 5.573, p < .001; see Fig 3). When the video ended 100 ms after the reorientation point of the kick (easy decision) a similar pattern was observed. In this case, if the simulated grunting was present, participants were 56 ms slower to respond to the direction of the kick (301 ms, SE = 8.7, vs. 245 ms, SE = 7.4; t(21) = 4.68, p < .001).

**Fig 4:**
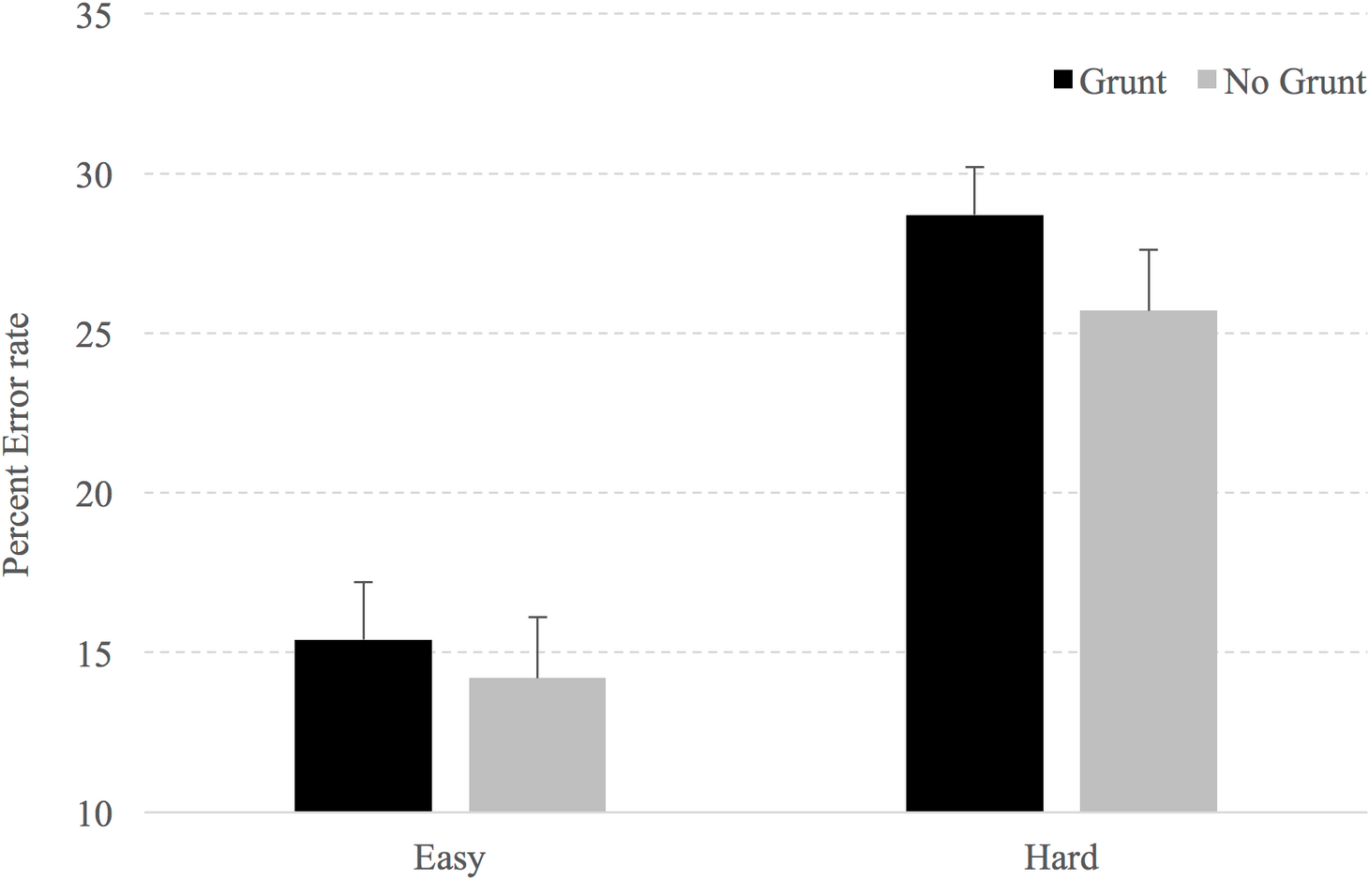
Error rates for easy and hard conditions. Error bars represent standard error of the mean.

The error data revealed a main effect of Difficulty, F(1,21) = 70.5, p < .001, with respondents making significantly more errors on hard than easy trials (27.2% vs. 14.8% respectively). Despite a numerical trend in the direction of more errors being made in trials with the simulated grunt (22.1% vs. 20%), a main effect for simulated grunting was not observed, F(1,21) = 2.95, p = .101, nor was an interaction observed, F(1,21) = .952, p = .34. Planned comparisons revealed that when the simulated grunt was present and the video stopped at the moment of reorientation of the kick (hard decision), participants made 3% more decision errors (28.7%, SE = 1.8, vs. 25.7%, SE = 1.5; t(21) = 2.15, p = .043; see Fig 4). However, there was no significant difference for the error rate with the easy video clips when simulated grunting was present (Sound = 15.4%, SE = 1.9 vs. 14.2%, SE = 1.9; t(21) = .727, p = .475).

## General discussion

There are several important findings that merit discussion. Overall, the investigation conducted here suggests that grunting is advantageous in terms of not only generating increased force when kicking, but also as a means of distracting an opponent. The former point was exemplified by significantly greater force produced by our participants when kicking with the kiai as opposed to without. It should be pointed out, however, that as the kiai is typically used by martial arts practitioners, preventing the kiai in our experiment may have been unnatural for some of our participants. Incorporating a group of novice participants could provide converging evidence for our present conclusions, though it is clear that the current findings are in line with other investigations suggesting that grunting can increase grip strength [5, 6], and velocity in forehands and serves in tennis [2, 3].

The divide and conquer research approach utilized here allowed us to determine whether the simulated grunting used in our experiments masks an important signal during multisensory processing, or instead, if it simply distracts an opponent. In previous work using similar conditions to those used here, participants were slower and made more errors when responding to the direction of a tennis shot that included a simulated grunt [4]. However, it was not possible to determine the underpinning reason for the decline in performance when a simulated grunt was present. In the present study distraction is likely the only viable explanation, as the act of kicking does not involve multisensory signals that could be masked.

When directly comparing simulated grunt versus no grunt response conditions, all conditions returned significant differences save the proportion of errors made in the easy condition. More specifically, in the hard condition participants were on average 45 ms slower to respond to the orientation of the kick when the simulated grunt was present, and made more decision errors (3%). While the difference in errors is numerically small, it should be noted that this was statistically significant and, moreover, from a practical standpoint even a single judgment error in mixed martial arts could lead to the end of the competition. The increase in response time was similarly longer (56 ms) for the easy condition when the kiai was present, although no difference in decision errors was observed. While it is possible that the easy condition was not sufficiently sensitive in order to observe any differences in judgment errors, it should be noted that the nearly 20% delay in an opponent's response time to a kick accompanied by a grunt could lead to significant consequences at both the amateur and professional levels of mixed martial arts. Collectively the present findings indicate that simulated grunting distracted our participants, slowing down their ability to respond to the kick, and at times led to misjudgments of the direction of the kick. The extent to which these findings extend to more complex real-world situations is an important question for future investigation.

Aside from the theoretical importance of these findings, there are clear practical implications. Indeed, a conservative estimate of the time it takes for a kick to leave the ground till contact (i.e., the foot traveling from the ground to striking the opponent) is approximately 300 ms (derived from the kicks used in video clips of Experiment 2). The slowdown in reaction time is profound given that the average human reaction time is about the same as the time it takes to throw a kick [25]. The combination of increased force and slowed reaction time provides a competitor a significant advantage. Moreover, it should also be noted that because a martial artist’s kiai is far louder than the 60 dbs used here, our present findings represent an extremely conservative first-approximation of the effects of a simulated grunt. Finally, it should be noted that our findings pertain to naïve participants, and therefore control for the potential confound of experience/expertise. An exciting avenue for future research will be to determine the robustness of these findings with professional MMA participants in both the current (i.e., video based) format and potentially in a real-world setting.

It is also important to highlight that the procedure used here (Experiment 2) closely mirrors the methodology used in Sinnett and Kingstone [4]. That is, a binary judgement was made as to the direction of either a multisensory tennis shot (left or right of the screen) or a unisensory kick (up or down) accompanied by a simulated grunt or not. Furthermore, the findings across both studies are strongly convergent, given that participants in the present Experiment 2 were consistently slower and sometimes less accurate when responding to the direction of a kick when a simulated grunt was produced. Therefore, given these similarities between studies, and in terms of parsimony, it is highly likely that in Sinnett and Kingstone [4] distraction is the preferred explanation for the decline in performance that resulted from an opponent’s grunt. Nonetheless, it should be noted that the time course for both sports is different, with the tennis ball reaching an opponent at a much later time compared to a kick. That said, and acknowledging the many variables associated with live competition, our results suggest that the kiai would have the potential to distract an opponent.

In light of these findings an intriguing question arises regarding whether grunting should be considered cheating. The issue of grunting has led to heated debates amongst tennis professionals, the fans, and the media, with some, including tennis legend Martina Navratilova, claiming that grunting in tennis is akin to cheating [26]. It is our opinion that because grunting leads to increased force when kicking (or hitting a ball), then it is difficult to construe grunting as cheating, as it is a mechanism that enables a player to generate greater force. Moreover, while the distraction that accompanies the grunt further benefits the grunter, the fact that the grunt is used to create more force appears to remove the onus of responsibility from the grunter, and place the burden firmly on the opponent to develop ways to cope with the grunt.

Several years ago (2012) the Women’s Tennis Association (WTA) stated that they intended to implement new rules in an attempt to reduce the loudness of grunts. More recently, newly appointed WTA Chief Executive Steve Simon said that he has no immediate plans to implement new rules beyond the current hindrance regulation [27]. As a grunt can be used to increase force, one could argue that this is the correct decision. Convergent support for this conclusion is provided by the case of mixed martial arts. In this sport, it is very unlikely that the competitors, fans, or governing bodies would discuss the possible advantages of grunting. In fact, the kiai is actively taught and regarded as something that is not only positive, but expected. And not without reason, as the findings from this research provide for the first time empirical evidence that suggests that the kiai can be used to not only increase force, but also to distract an opponent; a potent combination.
